# The assessment of the optimal duration of early intervention with montelukast in the treatment of Japanese cedar pollinosis symptoms induced in an artificial exposure chamber

**DOI:** 10.3109/21556660.2012.728547

**Published:** 2012-10-09

**Authors:** Kazuhiro Hashiguchi, Kimihiro Okubo, Ken-ichiro Wakabayashi, Nobuaki Tanaka, Yukiko Watada, Kiyochika Suematsu, Minoru Gotoh

**Affiliations:** 1 Department of Otolaryngology, Futaba Clinic, TokyoJapan; 2 Department of Otolaryngology, Nippon Medical School, TokyoJapan; 3 Department of Otorhinolaryngology, Kitasato Institute Hospital, TokyoJapan; 4 Tanaka ENT Clinic, Tokyo, Japan; Department of Otorhinolaryngology, Tokyo Women’s Medical University Medical Center East, TokyoJapan; 5 Department of Otorhinolaryngology, Keio University School of Medicine, TokyoJapan; 6 Pharmaceutical Department, Medical Corporation Shinanokai, Samoncho Clinic, TokyoJapan

**Keywords:** Early intervention, Environmental exposure chamber, Japanese cedar pollinosis, Montelukast Clinical Trial Registration: UMIN Clinical Trials Registry number

## Abstract

**Objective:**

The study objective was to investigate the prophylactic efficacy of montelukast (MLK) 10 mg in suppressing seasonal allergic rhinitis (SAR) symptoms induced by Japanese cedar (JC) pollen and to determine how many days before exposure to JC in the artificial exposure chamber (OHIO chamber) would be optimal to start administration.

**Methods:**

This was a single-institution, double-blind, randomized placebo-controlled four-group parallel inter-group comparison study. Adult volunteers with JC pollinosis were divided into four groups: an MLK 7-day administration group (*n* = 27), an MLK 3-day administration group (*n* = 27), an MLK 1-day administration group (*n* = 26), and a placebo group (*n* = 26). The mean change in total nasal symptom scores (nasal obstruction, nasal discharge and sneezing) (TNSS) and each of the nasal symptom scores during exposure of JC pollen in the OHIO chamber were investigated.

**Results:**

The mean change in TNSS was significantly lower in the MLK treatment group, regardless of the number of days of administration, than in the placebo group (*p* = 0.0192). The results for the individual nasal symptoms showed that nasal obstruction was significantly suppressed in the 1-day administration group as compared with placebo (*p* = 0.0076), but no differences were found in sneezing score between any of the groups. For nasal discharge, we found a trend towards the effect clearing up after 3 days of administration. No serious adverse events were observed during the study.

**Conclusion:**

Although this study was acute and this artificial exposure model was conducted out of the pollen season, nasal symptoms that developed in the pollen exposure chamber, especially nasal obstruction, were significantly suppressed by starting oral administration of MLK 10 mg at least 1 day before exposure. These results suggest that prophylactic administration of MLK is effective and safe in the treatment of SAR.

## Introduction

The prevalence of seasonal allergic rhinitis (SAR) has been increasing over the last decade and now affects up to about 30% of the Japanese population^1^. Since the land-shape of Japan is long in the north–south direction and possesses four distinct seasons with different climates within the different regions, pollen from more than 4500 kinds of weeds and trees disperse all over the country. Although there are patients with pollinosis to each of these various pollens, Japanese cedar (JC) pollen is the cause of seasonal allergic rhinitis with the largest number of patients. A very large amount of JC pollen disperses over hundreds of kilometers from the beginning of February to late April^2^.

According to a recent epidemiological study, the prevalence of JC pollinosis is 26.5% and an approximately 10% increase has been observed over the past 10 years^1^. In addition, the decrease in the age of onset is marked in urban areas. An increase in the amount of JC pollen in the past decade has been cited as one reason for this.

JC pollinosis patients suffer from very severe nasal and ocular symptoms as a result of pollen dispersal over a 2.5-month period. These symptoms develop and steadily grow with daily pollen dispersion, and decreases patients' quality of life^3^.

Allergic rhinitis (AR) is a type I allergic disease, and its nasal symptoms consist of sneezing, nasal discharge and nasal obstruction. Immediate and delayed allergic reactions are associated with pro-inflammatory mediators such as histamine, proteases, cysteinyl leukotrienes (CysLTs), prostaglandins, and platelet activating factor, as well as infiltration of inflammatory cells. It has been suggested that not only histamine, but also CysLTs plays an important role in the pathogenesis of allergic rhinitis^4^. The actions of CysLTs released after allergen exposure in AR are mediated through the LT1 receptor, which include increased nasal vascular permeability and stimulation of mucous glands. The former produces edema formation in the nasal tissue which contributes to nasal obstruction and the latter increases mucous production and secretion which leads to rhinorrhea^5^.

Montelukast (MLK) is a cysteinyl leukotriene receptor antagonist (LTRA) that has been proved to be effective and tolerable in the treatment of SAR^6–8^. It significantly improves daytime and nighttime nasal symptoms as compared with placebo. According to a systematic review of randomized trials of the effect of LTRA monotherapy, its efficacy in the treatment of SAR is better than that of placebo and equivalent to antihistamines^9^.

Japanese guidelines for AR, developed by Japanese AR specialists, recommend treatment plans for patients with JC pollinosis in consideration of Japanese circumstances, one of which is administration of a second-generation antihistamine or LTRA before the start of pollen dispersal^1^. One clinical study investigating the efficacy of early intervention with LTRA in the prevention of JC pollen symptoms has shown the effectiveness of LTRA in reducing SAR symptoms throughout the JC pollen dispersal period^10^. However, there have been no studies on how many days before pollen dispersal it is optimal to start administration of this kind of drug.

The aim of this study was to assess the optimal duration of early intervention with MLK 10 mg in the treatment of allergen-induced symptoms in patients with JC pollinosis using a pollen exposure chamber (OHIO chamber).

## Methods

### Study population

Adult volunteers with JC pollinosis were recruited to participate in the study. The inclusion criteria were: presence of allergic symptoms, particularly nasal congestion, during the pollen season for at least 2 years, with scores of 2 or more on the specific IgE-antibody test (Uni-CAP RAST) for JC pollen; absence of any abnormal findings on physical examination.

The exclusion criteria were: subjects who have nasal diseases (nasal polyps and/or deviated nasal septum) or an infectious disease (acute rhinitis, chronic rhinitis, congestive sinusitis, atrophic rhinitis, chronic rhinosinusitis, and flu-associated rhinitis) that would interfere with evaluating the efficacy of the drug; subjects who have a systemic disease including asthma, hypertension, diabetes mellitus; subjects who had undergone surgery, including laser surgery for the purpose of treating AR within 6-months prior to the start of the study; a history of hypersensitivity to the study drug; pregnant women or breast-feeding women, and women who were hoping to become pregnant. The following medications were prohibited throughout the study periods: steroid injections, oral or intranasal steroids, antihistamines, all other LTRA, or tranquilizers, or topical decongestants.

Subjects who were considered ineligible by the physician in charge were excluded as well.

This study was conducted in conformity with good clinical practice guidelines and the Declaration of Helsinki of 1995 (as revised in Edinburgh in 2000). The study protocol was reviewed and approved by an independent institutional review board of the Shinanozaka Clinic (Tokyo, Japan). Informed consent was obtained from all subjects before participation in the study.

### Study design and protocol

This was a single-center, double-blind, randomized, placebo-controlled four-group parallel inter-group study conducted in the non-pollen season, from May to June 2010, during which time the subjects did not have SAR symptoms.

**Figure 1.**
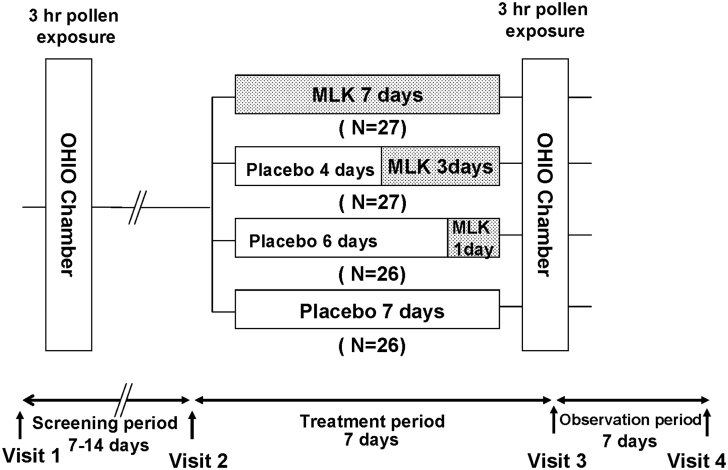
Study design. MLK, montelukast.

The study protocol is shown in the Figure 1. During screening and treatment periods, the subjects were exposed to JC pollen in a temperature- and humidity-controlled pollen exposure chamber (OHIO chamber)^11^. The OHIO chamber, located in the Samoncho Clinic in Tokyo, is a specialized room capable of accommodating a maximum of 14 persons that was set up for the purpose of dispersing a fixed concentration of JC pollen. Subjects are exposed to JC pollen at a concentration of 8000 grains/m^3^ for 3 hours, and were instructed to grade and record the severity of their nasal symptoms (sneezing, nasal discharge, nasal obstruction, and itchy nose) every 15 minutes according to the following 5-point scale: 0 = none (no symptoms); 1 = mild (symptoms present but easily tolerated); 2 = moderate (awareness of symptoms, bothersome but tolerable); 3 = severe (definite awareness of symptoms; difficult to tolerate but does not interfere with the activities of daily living); 4 = very severe (difficult to tolerate and interferes with the activities of daily living).

The total nasal symptom score (TNSS) was the sum of the subjects’ self-rated scores of three nasal symptoms (sneezing, nasal discharge, and nasal obstruction).

### Screening period

During the screening period, which was 2–4 weeks before the start of administration of the study drug, informed consent was obtained from the subjects, and they were examined to confirm that they met the inclusion criteria and did not fulfill any of the exclusion criteria. The screening exposure in the OHIO chamber was performed in order to ensure they would respond adequately to the pollen stimulation. The TNSS of the subjects during the last 1 hour was assessed. Subjects were enrolled in the study if their TNSS score was at least 3 and their score for nasal obstruction was at least 2 on one occasion.

### Treatment period

Administration of the study drug was commenced 14 days (±1 day) after the completion of the screening period. The actual drug consisted of capsules filled with MLK 10 mg and crystalline lactose. The placebo consisted of capsules containing crystalline lactose alone, whose external appearance was indistinguishable from that of the actual drug. The subjects were randomly divided into the following four groups who took one capsule daily at bedtime (from day 1 to day 7): a group that took MLK 10 mg for 7 days (7-day group); a group that took the placebo for 4 days and then took MLK 10 mg for 3 days (3-day group); a group that took the placebo for 6 days and then took MLK 10 mg for 1 day (1-day group); and a group that took placebo for 7 days (placebo group). Subjects were exposed to pollen in the OHIO chamber on the morning of the last medication (day 8). The exposure started at 9 a.m. The use of rescue drugs such as antihistamines, LTRA, systemic or intranasal steroids, and vasoconstrictor was not allowed during the treatment period.

### Endpoints

The primary efficacy endpoints were evaluated by comparing the changes from baseline in the TNSS of the subjects during the last hour of allergen exposure. The values before entering the chamber were used as the baseline values.

The secondary efficacy endpoints were the changes from baseline in each of the nasal symptom scores (nasal discharge, sneezing, and nasal obstruction) during the last hour of allergen exposure and changes in the volume of nasal discharge and number of sneezes. The amount of nasal discharge was measured by collecting previously weighed facial tissues every 30 minutes. The weight difference between the tissue before and after use was considered the amount of nasal discharge (g). The number of sneezes was counted and self-reported by each subject every 15 minutes.

### Safety

Safety was evaluated throughout the study period. All subjects were asked about their health status and adverse reactions during this period.

### Statistical analysis

For analyzing the backgrounds of the four treatment groups, we used an analysis of variance (ANOVA) for age, an IgE titer, the disease duration and the TNSS at screening period, and a chi-square analysis of the sex ratio. Values of *p* < 0.05 were considered to be statistically significant.

Analysis of the primary efficacy endpoint, the prophylactic efficacy of MLK, was performed using the maximum contrast method^12^ in order to analyze the dose–response patterns of medication days in the mean change in TNSS scores during the last hour of allergen exposure. To explore the dose–response pattern, the contrast coefficient vectors of administration [placebo, 1-day, 3-day, and 7-day] were set as follows: (1) a linear [3 1 −1 −3], (2) plateau by 3-day administration [5 1 −3 −3], and (3) plateau by 1-day administration [3 −1 −1 −1]. The error covariance was estimated with a repeated-measure analysis of covariance model with the baseline score as the covariate. The analysis model was included in the interaction between administrations and time point. *P*-value was calculated with adjustment of multiplicity by concurrent analysis of three patterns. The significance level was set at the one-sided 2.5%. The differences between the placebo group and the active treatment groups had been confirmed beforehand after calculation of the average of the mean TNSS scores during the last hour of allergen exposure and the two-sided 95% confidence interval (CI). The same method was applied to analyze the mean changes in the individual nasal scores (sneeze, nasal discharge, and nasal obstruction).

In the comparison of treatment groups, the statistical superiority of each of the three treatment groups (7-day, 3-day, or 1-day) compared with placebo was analyzed by following a hierarchy of conditions (step-down procedures). First, the 7-day administration group was compared with placebo (unpaired *t*-test, values of *p* < 0.05 were considered to be statistically significant). If the 7-day administration was superior to placebo, the second analysis set was performed. In the second set, the 3-day administration group was compared with placebo, in the same manner as in the first step. If the 3-day administration was superior to placebo, the third analysis set was be performed. In the third analysis set, the 1-day administration group was compared with placebo, in the same manner.

The analyses were performed using SAS for Windows Ver.8.2 (SAS Institute, Inc., Japan).

## Results

### Study subjects

Out of a total 230 subjects who were screened for eligibility, 106 subjects (45 males and 61 females), who satisfied the inclusion criteria and did not have exclusion criteria, completed the study. They were randomly assigned to the three MLK treatment groups (7 -, 3 -, and 1-day administration groups; *n* = 27, 27, and 26, respectively) and the placebo group (*n* = 26). There were no drop-outs in this study. The background of the subjects and their baseline characteristics are shown in Table 1. There were no significant differences among the four groups with respect to age, gender, duration of SAR, specific IgE titer, and the TNSS in the screening period. Most of the subjects showed mild-to-moderate nasal symptoms as assessed by allergy diaries kept during the pollen season.

**Table 1.** Demographic and baseline characteristics.

	Placebo	1-day	3-day	7-day
Number of subjects	26	26	27	27
Age (years)*	41.0 ± 2.2	41.9 ± 2.2	38.5 ± 1.7	36.5 ± 1.4
Gender				
Male	9	6	14	16
Female	17	20	13	11
Duration of SAR (years)*	13.4 ± 1.3	19.3 ± 1.8	16.8 ± 1.7	17.5 ± 1.6
Specific IgE titer*	3.77 ± 0.19	3.88 ± 0.19	3.70 ± 0.12	3.56 ± 0.18
TNSS in screening*	0.05 ± 0.03	0.10 ± 0.04	0.11 ± 0.05	0.14 ± 0.04

*Data are presented as mean ± SD. There were no significant differences among the four treatment groups. 1-day = 1 day administration of montelukast; 3-day = 3 days’ administration of montelukast; 7-day = 7 days’ administration of montelukast; TNSS = total symptom score.

### Efficacy

Table 2a shows the mean changes from the baseline in the TNSS and individual symptom scores during the last hour of exposure and 95% CI; Table 2b shows the dose–response relationships of them.

**Table 2a.** Average symptom scores during the last one hour of pollen exposure.

Treatment group	Placebo	1-day	3-day	7-day
	(*n* = 26)	(*n* = 26)	(*n* = 27)	(*n* = 27)
TNSS				
Mean ± SE	3.17 ± 0.20	2.27 ± 0.16	2.01 ± 0.16	2.31 ± 0.17
95% CI	2.78–3.56	1.95–2.59	1.70–2.33	1.98–2.64
vs. placebo	—	*p* < 0.05	*p* < 0.05	*p* < 0.05
Nasal obstruction				
Mean ± SE	1.29 ± 0.08	0.81 ± 0.07	0.67 ± 0.07	0.92 ± 0.07
95% CI	1.13–1.45	0.66–0.95	0.54–0.80	0.77–1.06
vs. placebo	–	*p* < 0.05	*p* < 0.05	*p* < 0.05
Nasal discharge				
Mean ± SE	1.42 ± 0.08	1.15 ± 0.07	0.95 ± 0.07	1.12 ± 0.07
95% CI	1.26–1.57	1.00–1.29	0.82–1.08	0.98–1.26
vs. placebo	–	*p* < 0.05	*p* < 0.05	*p* < 0.05
Sneezing				
Mean ± SE	0.46 ± 0.07	0.32 ± 0.05	0.39 ± 0.06	0.27 ± 0.05
95% CI	0.32–0.60	0.21–0.42	0.27–0.51	0.18–0.37
vs. placebo	–	N.A.	N.A.	N.S.

TNSS = Total nasal symptom score; 1-day = 1 day administration of montelukast; 3-day = 3 days’ administration of montelukast; 7-day = 7 days’ administration of montelukast; N.A. = not applicable; N.S. = not significant. *p*-value by unpaired *t*-test.

**Table 2b.** The dose response relationship in the mean changes in scores.

Analytic factor	Contrast coefficient	*t*-value	*p*-value
TNSS*			
	[3 1 −1 −3]	1.770	–
	[5 1 −3 −3]	2.174	–
	[3 −1 −1 −1]	2.341	0.019**
Nasal obstruction			
	[3 1 −1 −3]	1.794	–
	[5 1 −3 −3]	2.396	–
	[3 −1 −1 −1]	2.704	0.0076**
Nasal discharge			
	[3 1 −1 −3]	1.482	–
	[5 1 −3 −3]	1.854	0.0565
	[3 –1 −1 −1]	1.798	–
Sneezing			
	[3 1 −1 −3]	1.106	–
	[5 1 −3 −3]	0.933	–
	[3 −1 −1 −1]	1.173	0.1853

*The maximum contrast method is a statistical procedure to analyze dose–response pattern for biomedical or toxicological studies. For the suppression pattern of TNSS, it’s selected contrast was [placebo, 1-day, 3-day, 7-day: 3, −1, −1, −1] (*p* = 0.019). It was shown statistically significant difference that an effect served 1-day medication and reached the plateau. **p < 0.025. TNSS = Total nasal symptom score.

**Figure 2.**
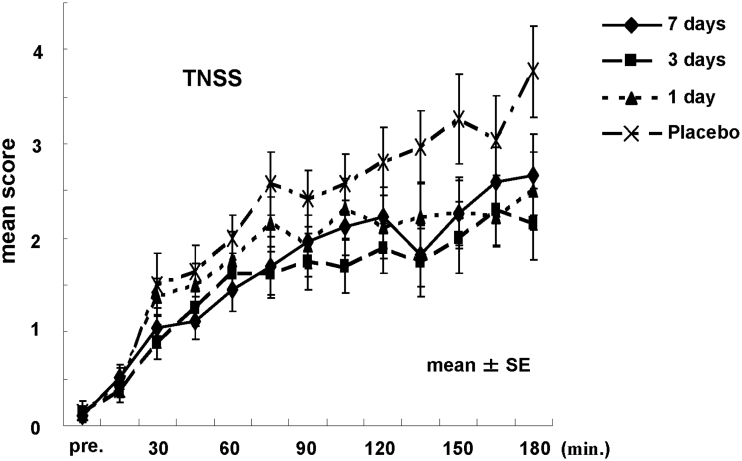
Time course of the total nasal symptom score (TNSS).

The average score in TNSS and 95% CI (in parentheses) was 3.17 (2.78–3.56), 2.27 (1.95–2.59), 2.01 (1.70–2.33), and 2.31 (1.98–2.64) for the placebo, 1-day, 3-day, and 7-day administration groups, respectively, suggesting that there were differences in the efficacy of suppression of nasal symptoms between placebo and active treatment groups. According to this analysis, the dose–response patterns evaluated by the maximum contrast method indicated that the selected contrast pattern was [3 −1 −1 −1] with a *p*-value of 0.019, indicating that the maximum effect for the suppression of nasal symptoms was obtained and reached plateau by starting the administration of MLK at least 1 day before exposure. Figure 2 shows the mean changes in TNSS score at each measurement point in the treatment groups. Compared with the placebo group, active treatment groups showed lower TNSS scores during pollen exposure.

**Figure 3.**
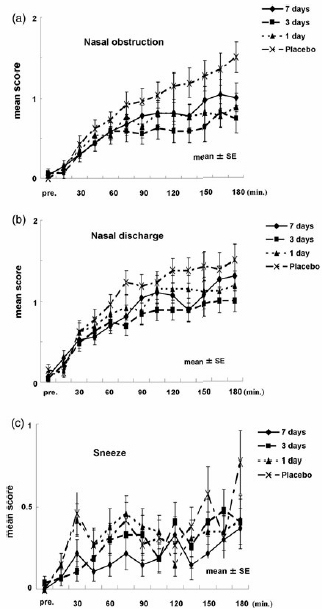
Time course of the nasal obstruction score (a), nasal discharge score (b) and sneezing score (c).

When the individual symptoms were evaluated, the differences in efficacy between the placebo and active treatment groups were confirmed from the mean scores and 95% CIs. For nasal obstruction and nasal discharge, the mean changes in the scores showed a similar pattern to those in the TNSS (Figure 3a,b). The selected contrast for nasal obstruction was [3 −1 −1 −1] with a *p*-value of 0.0076, demonstrating that the drug effect reached plateau after 1 day of administration. For nasal discharge, the selected contrast pattern was [5 1 −3 −3]. Although significant differences were not found among the treatment groups (*p* = 0.0565), it was suggested that the trend toward effects reached plateau by 3 days’ administration. For sneezing, no clear tendency was found in the mean change in scores of the treatment groups (*p* = 0.1853) (Figure 3c). Since a dose–response pattern could not be presumed, no significant differences were found among the treatment groups.

No significant differences were found among the groups in the amounts of nasal discharge and the number of sneezes (Table 3).

**Table 3.** Results of nasal secretion weight and number of sneezes.

	Placebo	1-day	3-days	7-days
Nasal secretion weight (g) (mean ± SD)*				
0–60 min	1.4 ± 1.7	1.2 ± 2.0	0.8 ± 0.9	1.1 ± 2.1
61–120 min	3.0 ± 3.6	2.5 ± 3.2	1.9 ± 2.4	2.6 ± 3.1
121–180 min	4.0 ± 5.0	3.0 ± 3.5	2.2 ± 3.0	2.9 ± 3.3
Numbers of sneezes (mean)*				
0–60 min	1.5	1.7	1.2	1.0
61–120 min	1.7	2.0	2.0	1.7
121–180 min	3.4	2.0	1.9	2.3

*There were no significant differences between the four treatment groups in nasal secretion weight and number of sneezes at any measurement point.

### Safety

No serious adverse events were observed in this study. Ten adverse events were documented, of which mild drowsiness in two subjects were regarded as being possibly related to MLK. All of the adverse events were mild and were resolved without any medication.

## Discussion

This study was conducted to investigate how many days from JC pollen dispersal to the start of administration of LTRA is sufficient in order to suppress symptoms induced in an artificial pollen exposure chamber. Nasal and ocular symptoms induced by constant but high concentrations of pollen for a short duration in the strictly controlled chamber are the result of an acute allergic reaction, whereas symptoms elicited during the natural pollen season are the result of the repeated exposure of pollen over a long duration^13^. However, a recent study demonstrated that symptoms elicited in the chamber are similar to those observed during the pollen season^14^. Thus this study model was considered to be suitable one. The maximum contrast method is a statistical procedure first introduced to analyze dose–response patterns for biomedical or toxicological studies to determine the minimum effective dose in clinical trials, the minimum toxic dose in toxicity studies, or a plausible dose–response pattern in dose–response studies^11^. We applied this method to investigate the optimal duration of administration of the study drug in the prevention of SAR symptoms. The study demonstrated that significant suppression in TNSS during exposure of pollen were observed in the MLK-treated groups, regardless of the number of days of administration, as compared with the placebo group. As for individual symptoms, nasal obstruction and nasal discharge but not sneezing were suppressed in MLK-treated groups and showed a similar pattern to the TNSS profile. Interestingly, the effect for nasal obstruction was shown to be significantly lower in the MLK groups, irrespective of the number of days of administration, compared to the placebo group. These findings indicate that administration of MLK 10 mg at least 1 day prior to pollen exposure reduces the development of nasal symptoms in the subjects with JC pollinosis.

Several studies have been published evaluating the clinical efficacy and safety of MLK monotherapy or combined with antihistamine in the treatment of SAR under natural pollen exposure^6,7,15,16^. According to a systematic review and meta-analysis of randomized studies, treatment with MLK for 2–4 weeks reduced daytime and nighttime nasal symptoms, rhinoconjunctivitis quality of life, and eye symptoms compared with placebo^9,17^. On the other hand, an environmental exposure unit (EEU) study in which the efficacy of MLK was evaluated and compared with levocetirizine in the subjects with SAR^18^, treatment with MLK showed significant improvement in the nose and eye symptoms, especially in nasal obstruction, as compared with placebo. Also, it showed an early onset of action of MLK after 1.5 hours of administration and remained effective for 24 hours.

In the present study, the effects of MLK on individual nasal symptoms showed that there was no significant difference in sneezing, but it suppressed nasal discharge and nasal congestion during pollen exposure and a significant difference in nasal obstruction was found in the last hour of exposure. CysLts have multi-functional properties in the pathogenesis of AR^5^. Direct challenge of LTD4 on the nasal mucosa in patients with AR significantly increased nasal secretions within 5 minutes with increased nasal mucosal blood flow and nasal resistance^19,20^. CysLts also increased vascular permeability which results in nasal obstruction^19^, a symptom which causes a deterioration in QOL the most in the patients with SAR. Since LTRA does not stimulate the sensory nerves^5^, sneezing seems not to be a correlated symptom. Although treatment with MLK showed significant improvement in sneezing in clinical studies of patients with AR^7^, its mechanisms have not been clearly demonstrated. Our findings, that a significant suppression of nasal obstruction and discharge in MLK groups was observed compared with that in placebo, appears to be consistent with the pharmacological features of this drug^21^.

Reduction in the number of eosinophils is known to be another pharmacological action of LTRA^22^. After treatment with MLK in patients with mild asthma, a significant decrease in the number of eosinophils was observed in a randomized, double-blind crossover study^23^. Thus, MLK taken several days in advance suppresses the infiltrations of inflammatory cells, especially eosinophils, into the nasal mucosa, which may be attributable to a reduction in the nasal obstruction.

Treatment with a second-generation antihistamine just before pollen dispersal has been popular in Japan in the management of SAR and its ability to suppress the symptoms or delay the onset of symptoms has been superior to treatment which was started after the appearance of SAR symptoms during the pollen dispersion period^1^. Several studies published in Japanese journals have confirmed the prophylactic effects of antihistamines^24^. In contrast, there have been only two studies of LTRAs in the prophylactic treatment of SAR compared with placebo or antihistamine^10,25^. Both randomized controlled studies were conducted with grass/cereals pollinosis or JC pollinosis patients under natural pollen exposure. One study, in which the number of participants was not large enough, showed that cetirizine or MLK monotherapy administered 6 weeks prior to the beginning of pollen season did not show significant improvement in rhinitis symptoms, whereas the treatment with combined these two drugs was effective in preventing in-season symptom^25^. The other study conducted in Japan, however, showed that LTRA started before the pollen season was significantly effective in reducing SAR symptoms and deterioration of QOL as compared with placebo^10^. In the present study, using a JC pollen exposure chamber, MLK administered prior to pollen exposure significantly suppressed allergen induced symptoms. Since limited data are available regarding the prophylactic efficacy in the treatment of SAR with LTRA, a study in large number of participants would be needed in a clinical setting.

Onset of action of MLK in SAR under natural pollen exposure has been investigated in double-blind placebo controlled trials. The results of these showed that significant relief in daytime and nighttime symptoms can been achieved starting on day 2^26^. Although our study is an acute environmental exposure model, the results showing suppression of symptoms in response to administration of MLK for at least 1 day appear to be valid. The short duration of taking drugs before pollen dispersal would be beneficial to patients with SAR because of the large quantity of JC pollen which disperses for a long time period.

## Conclusion

In conclusion, MLK 10 mg treatment started 1 or more days in advance before pollen exposure significantly suppressed the nasal obstruction and discharge induced in the OHIO chamber. This study is the first one to investigate assessing the preventive effects of MLK using a pollen exposure chamber. Our results indicate that SAR due to JC pollen would be more effectively controlled by pre-seasonal treatment with MLK in a clinical settings.

## Notes

**Notice of Correction** The version of this article published online on 18th October 2012 contained a typographical error on page 45. The error has been corrected for this version.
